# Ivabradine Reduces Chemokine-Induced CD4-Positive Lymphocyte Migration

**DOI:** 10.1155/2010/751313

**Published:** 2010-12-05

**Authors:** Thomas Walcher, Peter Bernhardt, Dusica Vasic, Helga Bach, Renate Durst, Wolfgang Rottbauer, Daniel Walcher

**Affiliations:** Department of Internal Medicine II-Cardiology, University of Ulm, Robert-Koch-Strareß8, 89081 Ulm, Germany

## Abstract

*Aims.* Migration of CD4-positive lymphocytes into the vessel wall is a critical step in atherogenesis. Recent data suggest that ivabradine, a selective I(f)-channel blocker, reduces atherosclerotic plaque formation in apolipoprotein E-deficient mice, hitherto nothing is known about the mechanism by which ivabradine modulates plaque formation. Therefore, the present study investigated whether ivabradine regulates chemokine-induced migration of lymphocytes. *Methods and results.* Stimulation of CD4-positive lymphocytes with SDF-1 leads to a 2.0 ± 0.1 fold increase in cell migration (*P* < .01; *n* = 7). Pretreatment of cells with ivabradine reduces this effect to a maximal 1.2 ± 0.1 fold induction at 0.1 *µ*mol/L ivabradine (*P* < .01 compared to SDF-1-treated cells, *n* = 7). The effect of ivabradine on CD4-positive lymphocyte migration was mediated through an early inhibition of chemokine-induced PI-3 kinase activity as determined by PI-3 kinase activity assays. Downstream, ivabradine inhibits activation of the small GTPase Rac and phosphorylation of the Myosin Light Chain (MLC). Moreover, ivabradine treatment reduces f-actin formation as well as ICAM3 translocation to the uropod of the cell, thus interfering with two important steps in T cell migration. *Conclusion.* Ivabradine inhibits chemokine-induced migration of CD4-positive lymphocytes. Given the crucial importance of chemokine-induced T-cell migration in early atherogenesis, ivabradine may be a promising tool to modulate this effect.

## 1. Introduction

Atherogenesis is an inflammatory process in the vessel wall involving inflammatory cells like monocytes, macrophages, and CD4-positive lymphocytes [1, 2]. In early atherogenesis, CD4-positive lymphocytes are attracted by chemotactic proteins such as RANTES and SDF-1 and enter the vessel wall as naïve TH0 cells. In the subendothelium, these cells then encounter antigens like oxidized LDL and differentiate into TH1 cells, subsequently releasing proinflammatory mediators like TNF-*α* and Interferon-*γ* (IFN*γ*). These cytokines then govern the inflammatory response in the vessel wall by activating other cells such as endothelial cells, macrophages, and vascular smooth muscle cells, thus promoting the inflammatory process in atherogenesis. Moreover, experimental studies have shown that a reduction in CD4-positive lymphocyte recruitment hampers lesion development and plaque formation [3, 4]. Still, most of these studies targeted the effect of T-cell-specific chemokines, but hitherto little is known about modulatory effects on CD4-positive lymphocyte migration.

Epidemiological studies have shown that elevated heart rate represents a risk factor for cardiovascular morbidity both in primary prevention and in patients with hypertension, coronary artery disease, and myocardial infarction [5–8]. Increased heart rate and reduced heart rate variability have been shown to be associated with coronary plaque rupture and subclinical inflammation in healthy middle-aged and elderly subjects [9, 10]. 

Selective heart rate (HR) reduction by I(f)-channel inhibition is a recently developed pharmacological principle in cardiovascular therapy. Among these newly identified HR-lowering drugs, only ivabradine has now become approved for clinical use. I(f)-channel inhibition mainly reduces HR, thereby improving myocardial oxygen supply, energy balance, and cardiac function. Ivabradine was well tolerated and revealed a good safety profile in the investigated study populations. 

Recent data have shown that treatment with ivabradine reduces oxidative stress, improves endothelial function, and prevents atherosclerosis in apolipoprotein E-deficient mice. In this study, ivabradine treatment leads to a reduction in oxidative stress and to a potent downregulation of MCP-1 expression in atherosclerotic plaques, causing less inflammation and decreased lesion size [11].

Nothing is known about the direct effect of ivabradine on chemokine-induced migration. 

Therefore, the current study examined the effect of ivabradine on CD4-positive lymphocyte migration and on intracellular signaling molecules involved.

## 2. Methods

### 2.1. Cell Culture

Human CD4-positive lymphocytes were isolated from freshly drawn blood of healthy volunteers by Ficoll-Histopaque (Sigma-Aldrich, Germany) gradient centrifugation to obtain mononuclear cells (PBMCs) and subsequent negative selection of CD4-positive T cells by magnetic bead separation (Miltenyi Biotec, Germany) as described by the manufacturer's protocol. The investigation conforms with the principles outlined in the Declaration of Helsinki and was granted by the university ethics review board. The purity of CD4-positive T cells was >97% as determined by flow cytometry.

### 2.2. In Vitro Cell Migration Assay

After isolation, CD4-positive cells were cultured in serum-free media for 16 h. T-cell chemotaxis was assayed under serum-free conditions in a 48-well microchemotaxis chamber (Neuroprobe, USA). Wells in the upper and lower chamber were separated by a polyvinylpyrrolidone-free polycarbonate membrane (pore size 5 *μ*m; Costar, Cambridge, MA). CD4-positive cells at a density of 5 × 10^5^/ml were pretreated for 15 min with ivabradine before 3 hours of incubation with SDF-1 or RANTES (Sigma-Aldrich, Germany). Migrated cells on the bottom face of the filter were stained and counted under the light microscope. Cells were counted in 5 random high-power fields per well (in control about 200–300 cells and in SDF-1 or RANTES-induced conditions about 600–700 cells per 5 random power fields per well).

### 2.3. Western Blot Analysis

 CD4-positive lymphocytes were left untreated or incubated at 37°C with 100 ng/mL RANTES (Sigma) or with 100 ng/mL SDF-1*α* (Upstate, Lake Placid, NY, USA) for times indicated. Cells were lysed in lysis buffer (50 mmol/L Hepes pH 7.4, 150 mmol/L NaCl, 1% (w/v) NP40, 1% (w/v) glycerol, 1 mmol/L MgCl_2_, 1 mmol/L MnCl_2_, 10 mmol/L NaF, 1 mM Na_3_VO_4_, 10 *μ*g/*μ*l aprotinin, 10 *μ*g/*μ*l leupeptin, 0.1 mmol/L PMSF). Aliquots of cell lysates were boiled in Laemmli buffer before running on SDS-PAGE. Immunoblotting was performed by running samples on SDS-PAGE with subsequent electrotransfer onto nitrocellulose membranes (Amersham Pharmacia Biotech, Amersham, England), blocking with 5% skim milk in TBS buffer with 0.1% Tween 20 for 1 h, and incubating with primary antibody anti-Rac1 was from Upstate (Lake Placid, NY, USA), anti-phospho-MLC2, anti-phospho-AKT, and anti-GAPDH were from Cell Signaling (Beverly, MA, USA), anti-*α*-tubulin was from Sigma and 1 : 2000 dilution of the secondary antibody (antigoat, antirabbit, or antimouse horseradish peroxidase (DAKO, Glostrup, Denmark)). Development was done by using enhanced chemiluminescence reagents (Pearce, Rockford, IL, USA) according to the manufacturer's specifications.

### 2.4. Phosphatidylinositol Kinase Assay

After isolation, human T cells were incubated for 16 hours in RPMI medium without serum. Cells pretreated for 15 min with or without ivabradine were stimulated with 100 ng/mL SDF-1. Standard PI 3-kinase activity assays were performed [12] using goat antihuman p85 (Santa Cruz) antibodies.

### 2.5. GTPase Activity Assays

For detection of GTP-bound Rac1, isolated CD4-positive lymphocytes were treated at 37°C with 100 ng/mL SDF-1 in presence or absence of ivabradine. Cells were lysed for Rac1-GTP pulldown experiments in 500 *μ*l Rac1-RIPA buffer (50 mmol/L Tris/HCl, pH 7.2, 150 mmol/L NaCl, 10 mmol/L MgCl_2_, 1% (v/v) Triton X-100, 0.5% (w/v) sodium deoxyholate, 10 *μ*g/*μ*l aprotinin, 10 *μ*g/*μ*l leupeptin, and 0.1 mmol/L PMSF). Cell lysates were cleared by centrifugation in a benchtop centrifuge at 15800 × g for 15 minutes at +4°C. Lysates were then incubated with 20–40 *μ*g glutathione S-transferase- (GST-) fusion proteins immobilized to Glutathione Sepharose 4B beads for 45 minutes at +4°C. The active GTP-bound form of Rac1 was determined by using a GST-PAK fusion protein. Beads were washed four times with washing buffer (50 mmol/L Tris/HCl, pH 7.2, 150 mmol/L NaCl, 10 mmol/L MgCL_2_, 1% (v/v) Triton X-100, 10 *μ*g/*μ*l aprotinin, 10 *μ*g/*μ*l leupeptin, and 0.1 mmol/L PMSF). Precipitated proteins were separated on 12% SDS-polyacrylamide gels, transferred to membrane, and detected by using appropriate antibodies.

### 2.6. ICAM3 Staining

Immunofluorescence staining was performed as described before [13]. In brief, 1-2 × 10^6^ CD-4 positive lymphocytes were incubated in special 4-well plates (Costar Corp., Cambridge, MA) in a final volume of 500 *μ*l complete medium on coverslips coated with collagen. Before treatment with SDF-1 (100 ng/mL, 30 min), cells were incubated with ivabradine for 15 min. Cells were then fixed with 3.7% (wt/vol) paraformaldehyde in PBS, pH 7.4, at room temperature and rinsed in TBS. Cells were incubated with a specific antibody against ICAM3 (mouse antihuman ICAM3, Caltag, UK) and after washing with PBS, carboxymethylindocyanine 3-Cy3-coupled (Dianova, Hamburg, Germany) goat antimouse IgGs were added as secondary antibodies (dilution 1 : 1,000) for 45 min. After washing again, slides were stained with DAPI (nuclei staining). Images were recorded with a fluorescence microscope (Leica, Wetzlar, Germany), and cells in 5 randomly chosen fields were analyzed. ICAM-3 translocation was considered to be present when a clear clustering of ICAM3 at the uropod was visible.

### 2.7. f-Actin Staining

CD4-positive lymphocytes (2 × 10^6^ cells/ml) were treated with SDF-1 at 100 ng/mL for times indicated after 15 min pretreatment with ivabradine. After stimulation, cells were fixed in 3.7% (wt/vol) paraformaldehyde in PBS, pH 7.4, and then washed at room temperature in TBS (50 mmol/L Tris-HCl, pH 7.6, 150 mmol/L NaCl, 0.1% NaN_3_). For intracellular staining of f-actin, cells were permeabilized with 0.1% Triton X-100 for 10 min before application of the first antibody. Cell suspensions were incubated for 30 min in PBS with FITC-conjugated phalloidin (Sigma no. P5282). Flow cytometry analysis was performed in an FACScan cytofluorometer (Becton Dickinson, Heidelberg, Germany), and induction of f-actin was measured.

### 2.8. Transendothelial Migration Assay

Migration of human CD4-positive lymphocytes across human endothelial cell monolayers was performed as previously described [14]. Briefly, human endothelial cells were cultured on gelatin-coated transwell filters. CD4-positive lymphocytes (0.5 × 10^5^ cells per well), pretreated for 30 minutes with and without ivabradine (0.05 or 0.1 *μ*mol/L), were added to the upper chamber of transwells inserts containing 1500 *μ*L HBSS. 100 ng/mL SDF-1 was placed in the bottom chamber to initiate transmigration. After 1-hour migration, cells that had transmigrated to the lower chamber were harvested and counted by flow cytometry.

### 2.9. Statistical Analysis

Results of the experimental studies are reported as mean ± standard deviation (SD). Differences were analyzed by 1-way ANOVA followed by the appropriate post-hoc test. A *P*-value <.05 was regarded as significant.

## 3. Results

### 3.1. Ivabradine Reduces SDF-1-Induced CD4-Positive Lymphocyte Migration

**Figure 1:**
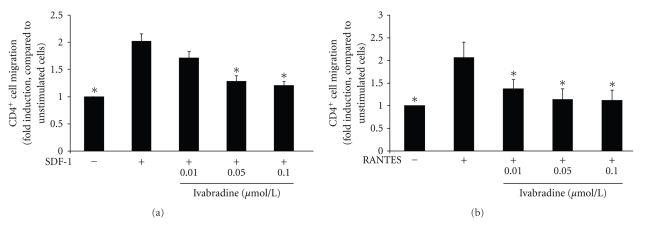
Ivabradine reduces SDF-1 and RANTES-induced CD4-positive lymphocyte migration. (a) Human CD4-positive cells were pretreated with ivabradine for 15 minutes at concentrations indicated before migration experiments using SDF-1 (100 ng/mL) were performed in a modified Boyden chamber. Data are expressed as fold induction compared to SDF-1-stimulated cells. Bars represent mean ± SD (*n* = 7); *P* < .01 compared to chemokine-stimulated cells. (b) Human CD4-positive lymphocytes were pretreated with ivabradine for 15 minutes at concentrations indicated before migration experiments using RANTES (100 pg/ml) were performed. Data are expressed as fold induction of chemokine-stimulated cells. Bars represent mean ± SD (*n* = 7); *P < .01 compared to chemokine-stimulated cells.

To examine the effect of ivabradine on CD4-positive lymphocyte migration, cells were stimulated with SDF-1 in the absence or presence of ivabradine, and lymphocyte migration was assessed in a modified Boyden chamber. SDF-1 treatment significantly induced cell migration by 2.0 ± 0.1-fold (*P* < .01; *n* = 7), and 15 min pretreatment of cells with ivabradine reduced this effect in a concentration-dependent manner to a maximal 1.2 ± 0.1-fold induction at 0.1 *μ*mol/L ivabradine (*P* < .01 compared with SDF-1-treated cells; *n* = 7) (Figure 1(a)).

### 3.2. Ivabradine Reduces RANTES-Induced CD4-Positive Lymphocyte Migration

Next, we examined the effect of ivabradine on RANTES-induced lymphocyte migration. Pretreatment with ivabradine for 15 min reduces RANTES-induced migration in a concentration-dependent manner to a maximal 1.1 ± 0.2-fold induction at 0.1 *μ*mol ivabradine (*P < .01 compared with RANTES-treated cells; *n* = 7) (Figure 1(b)). These results suggest that the effect of ivabradine on lymphocyte migration is independent of the stimulus employed.

Moreover, ivabradine did not affect cell viability and had no effect on the expression of the chemokine receptor CXCR4 as assessed by flow cytometry (data not shown).

### 3.3. Ivabradine Limits PI-3 Kinase Activity and Phosphorylation of AKT in CD4-Positive Lymphocytes

**Figure 2:**
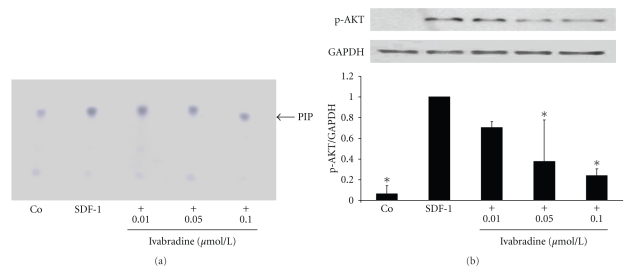
Ivabradine inhibits SDF-1-induced PI 3-kinase activity and phosphorylation of AKT. (a) Human CD4-positive cells were pretreated with ivabradine in different concentrations for 15 minutes before cells were stimulated with SDF-1 (100 ng/mL). After 5 minutes, PI 3-kinase activity assay was performed. Specific dots are labelled with an arrow (PIP). Three independent experiments showed similar results. (b) SDF-1 leads to phosphorylation of AKT. Isolated CD4-positive lymphocytes were pretreated with ivabradine in different concentrations indicated before stimulation with 100 ng/mL SDF-1 for 10 min. Total lysates were analyzed by immunoblotting employing antibodies against phospho-AKT. Equal loading of intact protein was confirmed by staining for GAPDH. Densitometric analysis were performed of 3 independent experiments. Data are expressed as p-AKT normalized to GAPDH. Bars represent mean ± SD. *P < .01 compared with SDF-1-stimulated cells; *n* = 3.

Activation of PI-3 kinase is a critical step in chemokine-induced T-cell migration downstream of the respective chemokine receptor [15]. Therefore, we examined the effect of ivabradine on PI-3 kinase activity. As demonstrated in Figure 2(a), ivabradine limited SDF-1-induced PI-3 kinase activity, suggesting that ivabradine modulates a very upstream step in the chemokine-activated signaling cascade.

Downstream of PI-3 kinase phosphorylation of AKT plays an important role in leucocyte migration [16, 17]. SDF-1 treatment significantly induced phosphorylation of AKT, and pretreatment with ivabradine reduced this effect in a concentration-dependent manner to a maximal 0.2 ± 0.1-fold induction at 0.1 *μ*mol ivabradine (*P < .01 compared with SDF-1-treated cells; *n* = 3) (Figure 2(b)).

### 3.4. Ivabradine Inhibits Activation of Rac1 and Phosphorylation of MLC

**Figure 3:**
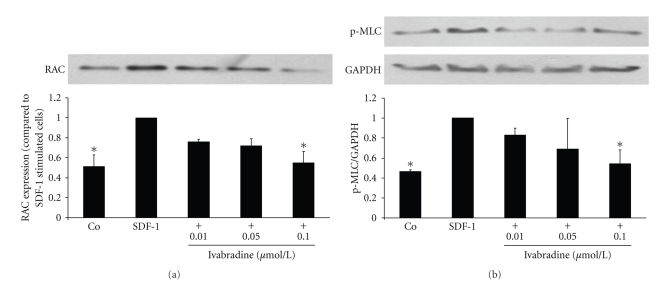
Ivabradine inhibits SDF-1-induced activation of Rac1 and reduces phosphorylation of MLC. (a) Human CD4-positive cells were pretreated with ivabradine (0.01, 0.05, and 0.1 *μ*mol/L) for 15 minutes before cells were stimulated with SDF-1 (100 ng/mL). After 3 minutes, GTPase activity assay was performed (affinity precipitation with GST-PAK). Densitometric analysis of 5 independent experiments. Bars represent mean ± SD. *P < .01 compared with SDF-1-stimulated cells. (b) SDF-1 leads to phosphorylation of MLC. Isolated CD4-positive lymphocytes were pretreated with ivabradine in different concentrations indicated before stimulation with 100 ng/mL SDF-1 for 5 min. Total lysates were analyzed by immunoblotting employing antibodies against phospho-MLC. Equal loading of intact protein was confirmed by staining for GAPDH. Densitometric analyses were performed of 5 independent experiments. Data are expressed as p-MLC normalized to GAPDH. Bars represent mean ± SD. *P < .01 compared with SDF-1-stimulated cells; *n* = 5.

Downstream of PI3K small Rho GTPases are important signaling molecules involved in leukocyte migration [18–20]. Therefore, we assessed the effect of ivabradine on Rac1 activity by performing affinity precipitation experiments with GST-PAK to which only the active GTP-bound form of Rac1 can bind. Stimulation with ivabradine diminished SDF-1-induced Rac1 activity in a concentration-dependent manner with a maximal effect at 0.1 *μ*mol/L (*P < .01 compared with SDF-1-treated cells; *n* = 5) (Figure 3(a)). 

At the uropod of the cell, phosphorylation of MLC is of critical importance for actin-myosin contractility [21]. Pretreatment of CD4-positive lymphocytes with ivabradine significantly reduced SDF-1-induced phosphorylation of MLC in a concentration-dependent manner to a maximal 0.5 ± 0.1-fold induction at 0.1 *μ*mol/L ivabradine (*P < .01 compared with SDF-1-treated cells; *n* = 5) (Figure 3(b)).

### 3.5. Ivabradine Reduces f-Actin Formation in CD4-Positive Lymphocytes

**Figure 4:**
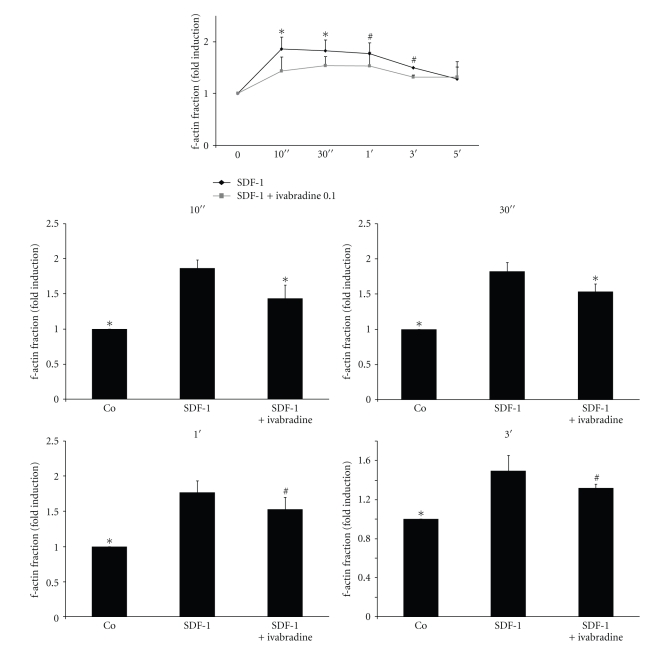
Ivabradine reduces actin polymerisation in CD4-positive lymphocytes. (a) Isolated lymphocytes were pretreated with ivabradine (*n* = 5) for 15 min before stimulation with SDF-1. Actin polymerisation was determined by flow cytometry at times indicated. Lower panel represents statistical analysis for the four timepoints indicated. Bars represent mean ± SD; *n* = 5; ^#^ *P* < .05; *P < .01 compared to SDF-1-stimulated cells.

Downstream of Rac1, f-actin formation is a crucial mechanism in cell polarisation during migration [22, 23]. Ivabradine significantly reduced SDF-1-induced f-actin formation as demonstrated by flow cytometry (Figure 4).

### 3.6. Ivabradine Inhibits ICAM3 Translocation in CD4-Positive Lymphocytes

**Figure 5:**
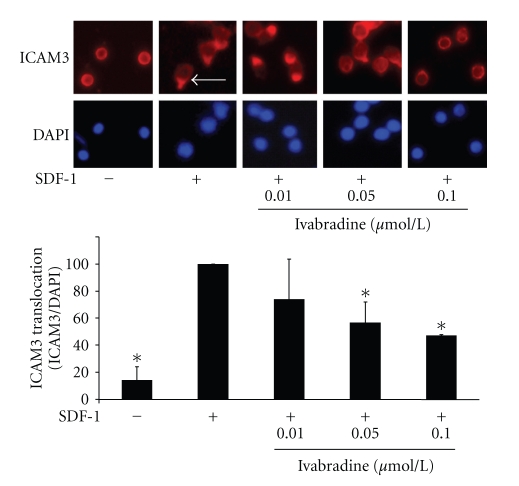
Ivabradine abolishes SDF-1-induced ICAM3 translocation. CD4-positive lymphocytes were pretreated with ivabradine (0.01, 0.05, or 0.1 *μ*mol/L) before stimulation with SDF-1 for 30 min. ICAM3 translocation was assayed using immunofluorescence staining. ICAM3 translocation at the uropod of migrating cells is indicated by the arrow. Lower panel shows statistical analysis of cells positive for ICAM3 translocation as % of DAPI-positive cells; bars represent mean ± SD; *n* = 6; *P < .01 compared to SDF-1-stimulated cells.

Next, we examined the effect of ivabradine on ICAM3 translocation at the uropod of migrating lymphocytes, a critical step in cell movement [24]. As shown in Figure 5, ivabradine significantly diminished SDF-1-induced ICAM3 translocation (Figure 5) with a maximal reduction at 0.1 *μ*mol/L.

### 3.7. Ivabradine Reduces SDF-1-Induced Transendothelial Migration of CD4-Positive Lymphocytes

**Figure 6:**
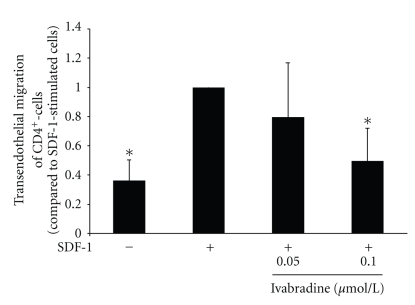
Ivabradine reduces SDF-1-induced transendothelial migration of CD4-positive lymphocytes. Human CD4-positive cells were pretreated with ivabradine for 15 minutes at 0.05 or 0.1 *μ*mol/L before migration experiments using SDF-1 (100 ng/mL) were performed in a transendothelial migration assay. Data are expressed as fold induction compared to SDF-1-stimulated cells. Bars represent mean ± SD (*n* = 6); *P* < .01 compared to chemokine-stimulated cells.

To facilitate the inhibitory effect of Ivabradine on SDF-1-induced migration of CD4-positive lymphocytes, we examined the effect of ivabradine on T cells in a transendothelial migration assay. Pretreatment with ivabradine in a concentration of 0.05 *μ*mol/L or 0.1 *μ*mol/L reduces SDF-1-induced transendothelial migration of CD4-positive lymphocytes (*P < .01, compared with SDF-1-stimulated cells, *n* = 6, Figure 6). 

## 4. Discussion

The present study demonstrates that ivabradine inhibits chemokine-induced migration of CD4-positive lymphocytes by reducing PI-3 kinase activity, phosphorylation of AKT, and activation of Rac-1 with subsequent inhibition of MLC-phosphorylation, f-actin formation, and ICAM3 translocation. 

Ivabradine exerts antianginal and anti-ischemic effects in patients with stable coronary artery disease. Clinical trials revealed improved exercise tolerance, increased time to exercise-induced ischemia, and reduced frequency of ambient angina attacks after I(f)-channel inhibition [25, 26]. Ivabradine given orally to mice reduces their heart rates without influencing left ventricular contractile function and therefore is used as a tool to study the effects of heart rate reduction in the vasculature. Ivabradine decreases markers of vascular oxidative stress, shown by reduced vascular NADPH oxides activity and decreased markers of lipid peroxidation. Furthermore, endothelial function was improved and atherosclerotic plaque formation was reduced in the ivabradine-treated group [11]. The content of inflammatory cells was not analyzed, especially the effect on chemokine-induced CD4-positive cell migration.

Chemokine-induced T-cell migration is mediated by activation of G-protein-coupled receptors leading to an increase in PI-3 kinase activity [15] with subsequent polarization of cells and formation of a leading edge and the so-called uropod in the rear [24]. The agonists employed in our study interfere with this signaling pathway by inhibiting PI-3 kinase activity and activation of Rac. Interestingly, 15 min pretreatment of CD4-positive cells with ivabradine already prevented the rapid effect of SDF-1 on PI 3-kinase activation, raising the question of a nontranscriptional effect of these agents in this context. This rapid effect described here was already seen for other nuclear receptors, like the PPAR*α* and PPAR*γ*. Previous studies from our own group showed similar results with PPAR*γ* -activating substances [13]. Downstream of PI 3-kinase and Rac1, ivabradine leads to a reduction in f-actin formation and ICAM3 translocation at the uropod of the cell, thus counterbalancing critical steps in cell movement [22–24]. In addition, the effect of ivabradine on cell migration does not depend on the chemotactic stimulus, since ivabradine diminished both SDF-1- as well as RANTES-induced lymphocyte migration. Further studies should show if this effect of ivabradine in vitro could be also observed in vivo and how this rapid response of ivabradine on chemokine-induced migration works. 

The importance of chemokine-induced migration and the chemokine receptor expression was shown in a mouse model with a genetic deletion of CCR5. In these ApoE−/− mice, the deletion of CCR5 protects from diet-induced atherosclerosis, associated with a more stable plaque phenotype, reduced mononuclear cell infiltration, Th1-type immune responses, and increased interleukin-10 expression [27]. Furthermore, blockade of MIF (macrophage migration inhibitory factor) but not of canonical ligands of CXCR2 or CXCR4 in mice with advanced atherosclerosis led to plaque regression and reduced monocyte and T-cell content in plaques [28].

The inhibition of lymphocyte migration shown here may influence atherogenesis at a critical step. CD4-positive cells, once recruited into the subendothelial space by chemokines like SDF-1 or RANTES, differentiate to TH1-cells, thus releasing proinflammatory cytokines which then promote inflammatory activation of other cells in the vessel wall [1, 2]. Therefore, modulating the migration of T-lymphocytes into the vasculature may target the inflammatory process in atherogenesis at a nodal point and as such modulate a critical step in atherosclerotic lesion development.
